# Effects of model choice, network structure, and interaction strengths on knockout extinction models of ecological robustness

**DOI:** 10.1002/ece3.4529

**Published:** 2018-10-31

**Authors:** Miranda S. Bane, Michael J. O. Pocock, Richard James

**Affiliations:** ^1^ Department of Physics and Centre for Networks and Collective Behaviour University of Bath Bath UK; ^2^ Centre for Ecology and Hydrology Crowmarsh Gifford Wallingford Oxfordshire UK

**Keywords:** coextinction, ecological interactions, extinction models, mutualistic network, network, plant–pollinator communities, pollinators, robustness

## Abstract

Analysis of ecological networks is a valuable approach to understanding the vulnerability of systems to disturbance. The tolerance of ecological networks to coextinctions, resulting from sequences of primary extinctions (here termed “knockout extinction models”, in contrast with other dynamic approaches), is a widely used tool for modeling network “robustness”. Currently, there is an emphasis to increase biological realism in these models, but less attention has been given to the effect of model choices and network structure on robustness measures. Here, we present a suite of knockout extinction models for bipartite ecological networks (specifically plant–pollinator networks) that can all be analyzed on the same terms, enabling us to test the effects of extinction rules, interaction weights, and network structure on robustness. We include two simple ecologically plausible models of propagating extinctions, one new and one adapted from existing models. All models can be used with weighted or binary interaction data. We found that the choice of extinction rules impacts robustness; our two propagating models produce opposing effects in all tests on observed plant–pollinator networks. Adding weights to the interactions tends to amplify the opposing effects and increase the variation in robustness. Variation in robustness is a key feature of these extinction models and is driven by the structural heterogeneity of nodes (specifically, the skewness of the plant degree distribution) in the network. Our analysis therefore reveals the mechanisms and fundamental network properties that drive observed trends in robustness.

## 1 INTRODUCTION

Network analysis has become an important tool for ecologists seeking to understand the vulnerability of ecosystems to natural and anthropogenic disturbance. Recent research has centered on network approaches for improving our understanding of plant–pollinator communities and extinctions, especially in the light of the widely documented declines in key insect pollinators such as honeybees, bumblebees, and butterflies (Benton, 2006; Biesmeijer et al., 2006; Goulson, Lye, & Darvill, 2008; Senapathi et al., 2015). These trends are concerning for biodiversity, ecosystem function, and food security (Potts et al., 2010) as insect pollinators play a vital role in providing ecosystem services (Bailes, Ollerton, Pattrick, & Glover, 2015). They feed on nectar and pollen provided by plant species, and whilst doing this, facilitate the fertilization of plants via cross‐pollination (Free, 1993; Lubbock, 1897). In plant–pollinator systems, the community can be regarded as a bipartite network comprising two distinct guilds of organisms in which each node represents a species, and species are connected by edges indicating interactions, which may be directly observed, indirectly observed (e.g., pollen analysis), or inferred (Morales‐Castilla, Matias, Gravel, & Araújo, 2015).

Models of community robustness based on observed plant–pollinator networks (available, e.g., from http://www.web-of-life.es and https://www.nceas.ucsb.edu/interactionweb/resources.html) usually fall into one of two types. In the first (see for example Bastolla et al., 2009; James, Pitchford, & Plank, 2012), the community is modeled as a dynamical system, in which the population of each species is affected by the interactions that species has with others. The dynamics are typically run to fixation, and the populations at fixation used to determine community robustness.

The second approach, adopted here, is to model the tolerance of the network to simulated extinctions (henceforth “knockout extinction models”). In ecology, this approach was applied first to multitrophic food webs (Dunne, Williams, & Martinez, 2002) and then mutualistic bipartite networks, especially plant–pollinator networks (Kaiser‐Bunbury, Muff, Memmott, Müller, & Caflisch, 2010; Memmott, Waser, & Price, 2004). Campbell, Yang, Shea, and Albert (2012) use a very similar approach to study the effects of forced species extinctions. The networks they analyze differ from those considered here, in that they are all generated by a (dynamic Boolean) model of plant–pollinator community formation (Campbell, Yang, Albert, & Shea, 2011).

**Figure 1:**
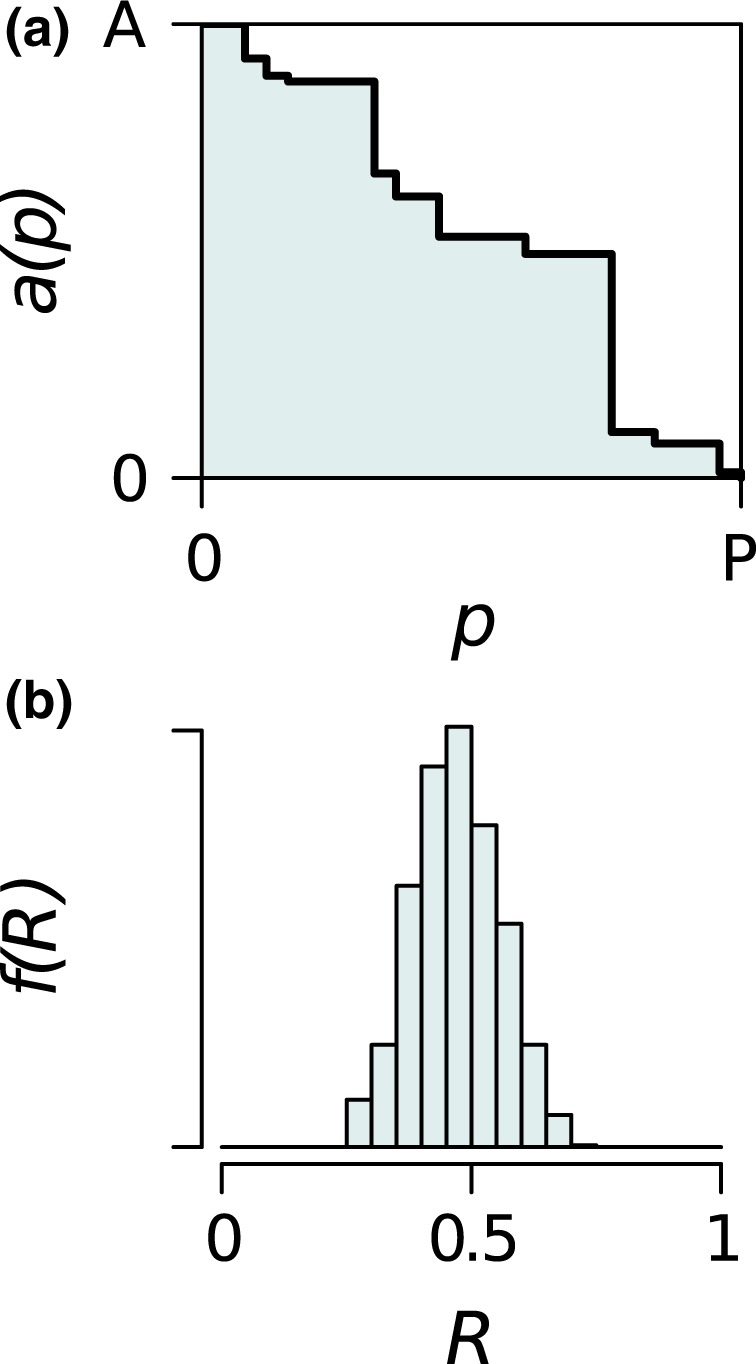
The output of a knockout extinction model. (a) For a single extinction sequence, the number of surviving pollinator nodes *a* reduces as the number of plant nodes made extinct, *p*, increases until *a* = 0. Robustness (*R*) = 0.550 is the area under *a*(*p*), divided by the area of the rectangle, *AP*. (b) In all our extinction models, the value of *R* depends on the order in which plants are made extinct, so many simulations of random sequences of primary extinctions are used to produce a distribution of robustness values *f*(*R*)

Knockout extinction models estimate the robustness of a plant–pollinator network by sequentially removing species of the primary type (e.g., plants) and recording the number of surviving species of the secondary type (e.g., pollinators), by applying some predetermined rule for species survival. Network robustness can then be determined from the area under the curve of the proportion of the secondary type that survive against the proportion of the primary type removed (Burgos et al., 2007; see Figure 1a).

In the simplest, “Secondary Only” (SO) knockout models, primary extinctions from one guild lead only to secondary extinction of species in the other guild. Primary extinctions are chosen in a specific order—determined by the number of interactions a species has, for example—or in a random order (Dunne et al., 2002; Memmott et al., 2004 and Pocock, Evans, & Memmott, 2012). A key development by Vieira and Almeida‐Neto (2015) was to allow coextinction due to feedback between guilds, so permitting cascades of extinctions. The propagating extinction model of Traveset, Tur, and Eguíluz (2017) incorporates empirically estimated dependencies of plants on pollinators. In a different development, Kaiser‐Bunbury et al. (2010) allowed edge rewiring (pollinators switching from one plant to another) based on empirical evidence; others have explored robustness to edge, not node, knockouts (Santamaría, Galeano, Pastor, & Méndez, 2016; Valiente‐Banuet et al., 2015).

SO models were used to show that the robustness of communities to random primary extinctions increases with network connectance, that is the fraction of the possible interactions that were actually observed (Dunne et al., 2002) and the resulting robustness was often interpreted in terms of network nestedness (Memmott et al., 2004). Vieira and Almeida‐Neto (2015) found that cascades were more likely in highly connected networks. However, more detailed investigation of the impact of network structure on robustness has been lacking.

Most early empirical plant–pollinator networks were binary; interactions between pairs of species were either observed or not. However, researchers are increasingly measuring the frequency or importance of interactions to create weighted networks, yielding a better description of the interactions observed (Ings et al., 2009 and Memmott, 1999), and accounting better for undersampling biases (Bersier, Banašek‐Richter, & Cattin, 2002). More recent models have used weighted data in different ways: using node abundance to weight the binary outcomes (Kaiser‐Bunbury et al., 2010) or using empirically determined, weighted dependences of plant species on pollinators (Traveset et al., 2017).

One of the features of knockout extinction models is that, when using random sequences of primary extinctions on a single empirical network, there is a broad distribution in the resulting robustness values (Figure 1b). Robustness must therefore be a product both of structural heterogeneity of the network (e.g., Pastor, Santamaria, Mendez, & Galeano, 2012) and of the method of producing extinction sequences.

The aim of this paper was to understand in detail which features of knockout models, and which properties of empirical ecological networks, are responsible for the central value and range of computed robustness distributions. To this end, we bring together a suite of models—a simple SO model and two simple propagating extinction models—and use them to compute the robustness of a number of empirical plant–pollinator networks in both binary and weighted form. The models were chosen for their simplicity and direct comparability, not to achieve ecological realism.

## 2 MATERIALS AND METHODS

In this study, we examine the robustness of observed plant–pollinator networks that describe observed interactions between species in a community. A network has *P* plant nodes and *A* animal nodes, and contains *E* interactions between species*,* encoded in the *A *× *P* matrix ***M***. Interactions may be binary (b) or weighted (w).

We illustrate our models and findings using a plant–pollinator network, collected by Memmott (1999), from Ashton Court, a site in Bristol, UK. We will refer to this as the Ashton Court (AC) network. This is a well‐sampled network (Blüthgen, Menzel, & Blüthgen, 2006) with interactions recorded over a short period of time (1 month). The AC network is highly resolved: all plants were identified to species (*P *=* *25) and pollinators (*A *=* *79) mostly identified to species level (morphotyped otherwise). ***M*** ^AC^ contains 104 species, *E *=* *299, with connectance (proportion of realized interactions) *c *=* *0.151 and nestedness (Almeida‐Neto, Guimarães, Guimarães, Loyola, & Ulrich, 2008) NODF = 42.5%. Interactions in the AC network are weighted by the number of observed visits of each pollinator species to each plant species. The plant degree distribution is highly skewed, with a high proportion of pollinators visiting a single plant species, as is often the case in plant–pollinator networks.

For comparison, we also present results for five other networks. We selected networks (summarized in Table 1) that had weighted edges (by visits), were well resolved, had *P *>* *12, had a range of *c* and NODF, and for which, we had access to collection methods.

**Table 1:** Summary of networks used in this paper

Network	Plants (*P*)	Pollinators (*A*)	Interactions (*E*)	Connectance (*c*)	Nestedness (NODF)	Largest P degree	Reference
Ashton Court	25	79	299	0.151	42.54	49	Memmott (1999)
Ottawa	13	34	141	0.319	40.96	18	Small (1976)
Mauritius	14	24	46	0.137	18.30	7	Kaiser‐Bunbury et al. (2010)
Shelfanger	16	36	85	0.148	35.66	21	Dicks, Corbet, & Pywell (2002)
Hickling	17	61	146	0.141	52.27	49	Dicks et al. (2002)
Creus	32	81	319	0.123	28.01	28	Bartomeus, Vilà, & Santamaría (2008)

### 2.1 Model development

**Figure 2:**
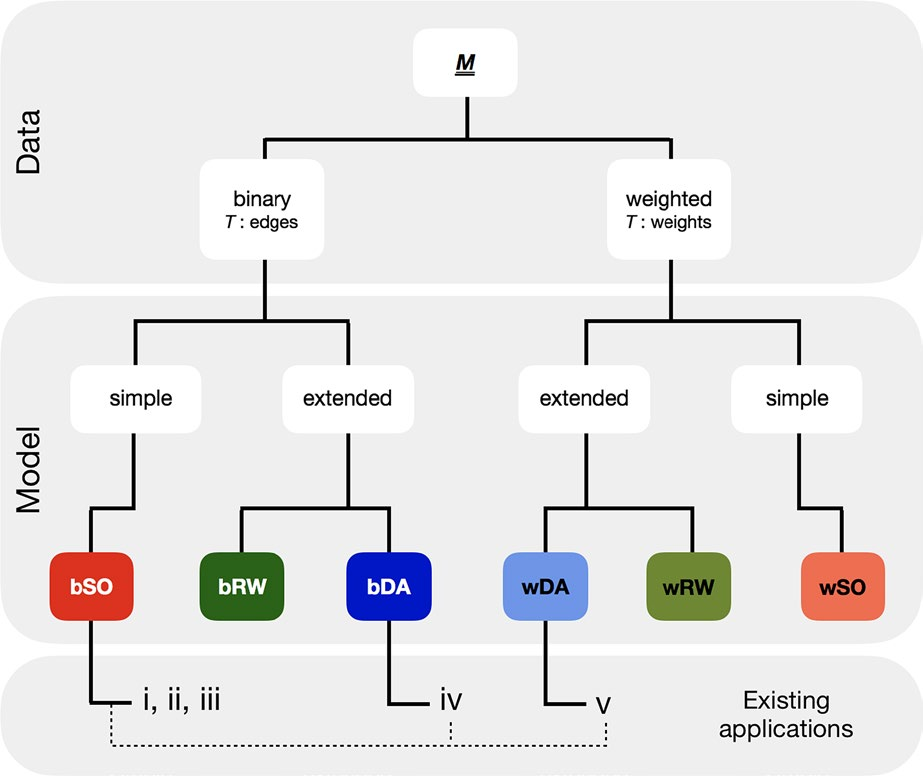
A framework of knockout extinction models, with those used in this paper highlighted in color. All models start from an observed mutualistic bipartite network ***M*** that can be binary (prefix b) or weighted (w). For binary data, the threshold *T* is applied to the number of edges; for weighted data, it is applied to the weights. Models are split into those that produce entirely random primary extinction sequences: Secondary Only (SO), and those that introduce other methods for determining extinction sequence: Deterministic Avalanche (DA) and Random Walk (RW). (i–v) indicate previous studies that used similar models, but where (i–iii) *T *=* *1: (i) Dunne et al., 2002; (ii) Memmott et al., 2004; (iii) Kaiser‐Bunbury et al., 2010; (iv) *T* is applied stochastically and extinctions can “cascade” (Vieira & Almeida‐Neto, 2015) and (v) a hybrid of (iv) and bSO with empirical plant dependencies (Traveset et al., 2017)

We took as our starting point the extinction model of Memmott et al. (2004), who analyzed the robustness of binary networks by making species of one type (in their case, pollinators) extinct in a random order, that is they used a random primary extinction sequence. From this, we developed two new extinction models, with differing ecological bases, that each includes subsequences of plant extinctions determined by network structure. All three models (summarized in Figure 2) use either edge weight or (binary) edge existence to decide secondary (and further) extinctions.

In this section, we first describe the features that are common to all our extinction models and then outline the distinctive features of each, providing the ecological context and highlighting the relationships between ours and previous knockout extinction models.

### 2.2 Universal model features

Starting from the observed matrix ***M***, a node of one guild (plants) is removed as a primary extinction. Extinctions result in the loss of interactions from ***M***, monitored in the “reduced” matrix ***C***. The loss of interactions may, according to the rules of the particular model, result in the secondary loss of nodes of the other guild (pollinators). In our new models (see below), the rules admit the possibility of each secondary pollinator extinction giving rise to further knock‐on plant extinctions, further pollinator extinctions, and so on. Any such plant extinctions cannot be considered “primary”, but will take their place in what we shall continue to refer to as a “primary extinction sequence” of the *P* plant species.

All models proceed until all plant nodes are removed and all species—plants and pollinators—are extinct, as in previous studies. The robustness (*R*) of the network is calculated as

(1) $$R=\frac{1}{\text{AP}}\sum_{p=0}^{P}a\left(p\right),$$

Where *p* is the number of plant species that have gone extinct (from 0 to *P*) and *a*(*p*) is the number of pollinator species remaining in the network (from *A* to 0). *R* is the normalized (0 < *R *<* *1) area under the curve of a graph of the proportion of plant nodes that have gone extinct against the proportion of surviving pollinator nodes (Figure 1a). Values of *R* closer to 1 indicate higher “robustness” of the network to primary extinctions (e.g. Burgos et al., 2007). We use *a*(*p*) as our response variable for all models in order to facilitate comparisons, although we note other options are possible: Kaiser‐Bunbury et al. (2010) used the sum of interaction weights *w*(*p*).

The value of *R* is dependent on the specific sequence of primary extinctions, so running many random extinction sequences will, for all our models, produce a frequency distribution of values of *R* (Figure 1b) which we denote *f*(*R*).

A key model feature we adopted, one that is necessary to put all models on an equal footing, and thereby to enable fair comparison between them, is a threshold rule for secondary extinctions: A node becomes extinct once it has lost a fraction *T* or more of its observed interactions (binary ***M***), or of its observed total interaction weight (weighted ***M***). Clearly, the value of *T* that we choose is arbitrary. It must lie in the range 0 < *T* ≤ 1. [*T *=* *0 is an uninformative case; all pollinator species become extinct after the first primary plant extinction; *T *=* *1 generates the extinction rule for most previous models (Dunne et al., 2002; Kaiser‐Bunbury et al., 2010; Memmott et al., 2004), although Vieira and Almeida‐Neto (2015) introduced a node‐specific threshold ≤1.] We generated distributions of robustness *f*(*R*) for a range of threshold values (0.1 to 1 at 0.1 intervals) for six observed plant–pollinator networks (summarized in Table 1) to determine the effect of *T* on *R*. We then chose a threshold of *T *=* *0.5 for all nodes for the remainder of this paper: That is, a secondary extinction occurs when a node has lost at least half its interactions (binary ***M***) or weights (weighted ***M***). It should be noted that the “effective threshold” (*T*_eff_) could be greater than *T*; for example, with a binary network and *T *=* *0.5, a node linked to five others would go extinct after losing three edges, giving an effective *T* of 3/5 = 0.6. Since most pollinators are observed visiting a relatively small number of plants, the difference between the specified and the “effective threshold” can be noticeable; we report the node‐averaged *T*_eff_ in all cases (Table S1).

### 2.3 New extinction model features

We present three distinct models, which we denote: 1. Secondary Only (SO), 2. Deterministic Avalanche (DA), and 3. Random Walk (RW). Each model can be used with binary or weighted interaction data and is prefixed with “b” or “w” to indicate which.

In ecological terms, SO is the most simple; the next (plant) extinction is always chosen randomly from those remaining, and all choices are independent of each other. The SO model is essentially that used by Dunne et al. (2002), Memmott et al. (2004) and others, and serves as our baseline. Its ecological premise is that a plant extinction will only affect the pollinator species that visit that plant; that is, there is a uni‐directional dependence in the interactions. The DA and RW models each introduce mutualistic dependencies between the guilds, in ways that remove independence from some subsequences of plant extinctions; that is, each allows the spread of extinctions through the community network. In DA, extinctions “ripple” out from an initial extinction causing a wave of collapse, as resources (interactions) diminish for both guilds. In RW, the contagion of extinction jumps from plant to plant according to their number of shared visitors, as might occur when a plant disease is spread through the community by visiting pollinators, or a pollinator disease is spread through shared floral resources (as reported by McMahon et al., 2015).

#### 2.3.1 Model 1. Secondary only model (bSO and wSO)

In the Secondary Only model, the order of primary plant extinctions is random. All pollinator extinctions are secondary and determined by the threshold rule. The method is as follows:

1. Select a random plant species (*e*) for primary extinction from those left (from matrix ***M*** the first time, then subsequently matrix ***C***)
2. Make pollinator species connected to *e* extinct if they have lost a proportion ≥*T* of their original edges (bSO) or edge weights (wSO)
3. Count the number of pollinator species remaining, *a*(*p*), in the updated network (matrix ***C***)

Repeat steps 1 to 3 until there are no species remaining. Then calculate *R* according to Equation 2.

In the special case *T *=* *1, the bSO and wSO models are identical to each other, and to the model described by Memmott et al. (2004). Kaiser‐Bunbury et al. (2010) employed an adaptation to the special case *T *=* *1 and used the weight of remaining edges *w*(*p*) as their response variable.

#### 2.3.2 Model 2. Deterministic avalanche model (bDA and wDA)

In the DA model, a randomly chosen primary (plant) extinction—a “trigger”—may produce secondary extinctions (of pollinators) that themselves leave plant species with less than a fraction *T* of their observed interactions. If this happens, there is an “avalanche” of plant extinctions. During the avalanche, the sequence of plant extinctions is not random, but is determined by network structure. At the end of an avalanche, a new, random, trigger is chosen. The method is as follows:

1. Select a random plant species (*e*) for primary extinction from those left (from ***M*** the first time, subsequently ***C***)—this is a trigger
2.
  - Make pollinator species connected to *e* extinct if they have lost a proportion ≥*T* of their original edges (bDA) or edge weights (wDA)
  - Count the number of pollinator species remaining, *a*(*p*), in ***C***
  - Make plant species (there may be more than 1) extinct according to the threshold rule as above

Repeat steps 2a to 2c until there is no further spread of extinctions, then repeat from step 1 with a new trigger

Repeat steps 1 and 2 until there are no species remaining in the network. Then calculate *R* according to Equation 2.

Were *T *=* *1 used here, step 2c would never result in tertiary plant extinctions and no avalanches would occur, so the DA and SO models would be identical. The “stochastic co‐extinction model” (SCM) developed by Vieira and Almeida‐Neto (2015) is a special case of the bDA model where the threshold is applied stochastically and is node specific; specifically, extinctions of nodes at our step 2c occur with probability = 1‐ (remaining interactions)/(interactions at start). We adopt the term “avalanche” for our spreading deterministic extinctions to differentiate them from the stochastic “cascades” of Vieira and Almeida‐Neto (2015), which occur once only, triggered by the first primary extinction. Traveset et al. (2017) employed what is essentially a hybrid SCM‐bSO model, with empirical dependencies for plants and allowing only two‐step cascades.

#### 2.3.3 Model 3. Random walk model (bRW and wRW)

The RW model is similar to DA, in that a trigger can cause an avalanche of nonrandom plant extinctions. In this model, the order of plant extinctions within an avalanche is determined by the (updating) structure of the *P* × *P* matrix ***F*** whose entry *F*_*eg*_ is the number of remaining pollinator species shared by plant species *e* and *g*. The full method is as follows:

1. Select a random plant species (*e*) for primary extinction from those left (from ***M*** the first time, subsequently ***C***)
2. Construct matrix ***F***
3. Select the next plant extinction (*f*) from ***F***. Each potential choice of plant (*g*) is chosen with a probability proportional to *F*_*eg*_.
4. Make pollinator species connected to *e* extinct if they have lost a proportion ≥*T* of their original edges (bRW) or edge weights (wRW)
5. Count the number of pollinator species remaining, *a*(*p*), in the updated matrix ***C***
6. Identify plant *f* as the new *e* and make it extinct
7. Loop through steps 2–6. If no neighbors exist in step 3, revert to step 1.

Repeat steps 1–7 until there are no species remaining in the network. Then calculate *R* according to Equation 2.

### 2.4 Natural extensions of our models

We have coded these three models for application with a random order of primary plant extinctions (i.e., the selection of the next extinction in step 1 of Models 1,2 and 3 is random). The models can all easily be modified to use ordered primary extinctions, where the choice of plant in step 1 is according to a predetermined rule (based on node degree, biological plant trait etc.). The models can also be applied to bipartite networks with uni‐directional dependencies (no feedback between the trophic levels, e.g., trophic or host–parasitoid interactions), though in that case avalanches cannot occur.

### 2.5 Comparison of robustness distributions from the three extinction models

The distribution *f*(*R*) generated from a single network ***M*** will depend on the model used and whether the edges are weighted or binary. If there are *P* plant species in the network, there are *P*! distinct plant sequences. The SO models sample uniformly from these possibilities (i.e., all sequences are equally likely). The DA and RW models do not sample uniformly, because avalanches produce nonrandom subsequences determined by the structure of the network. Using binary and weighted versions of the Ashton Court (AC) network, we generated 25,000 extinction sequences using each of the three models, in order to assess the effect of model choice on *R*. To create values of *R* that lie close to the theoretical maximum and minimum bounds, we ran bSO with plant extinctions in order of increasing and decreasing degree.

### 2.6 Testing on other networks

We tested our models on five other networks (Table 1). For each network, we generated 25,000 extinction sequences, using each of the three models, in binary and weighted form. We used a fixed threshold of *T* = 0.5 for all cases because we are not directly comparing the networks, only seeking to confirm the generalities of the resulting *f*(*R*) distributions.

### 2.7 Assessing how node and network‐level properties affect variation in robustness

The breadth of the distribution *f*(*R*) appears to be large in networks, such as AC, with a large range in plant degree (see Section 3). Previous studies have hinted at the probable role of degree (*k*) in determining robustness (James et al., 2012; Joppa, Montoya, Vicente, Sanderson, & Pimm, 2010; but see also Blüthgen et al., 2006). We therefore constructed two tests of the effect of degree on robustness, using the AC network as a test case, under each of our three knockout extinction models.

#### 2.7.1 Robustness distribution of networks with manipulated degree distributions

Our aim here was to look at the effect on *f*(*R*) of replacing one or both of the observed degree distributions (*g*_*A*_(*k*) for pollinators, *g*_*P*_(*k*) for plants) with something closer to what we would expect from random rewiring of the observed interactions; a Poisson‐like distribution with a well‐defined single peak, relatively small variance and few outliers.

Firstly, we constructed an ensemble of 10,000 networks in which all 299 interactions in the binary **M** ^AC^ were placed between a random plant and pollinator, enabling us to compute the randomized degree distribution (${\tilde{g}}_{A}\left(k\right),\;{\tilde{g}}_{P}\left(k\right)$ ) for each random network and the average (or expected) degree distributions (*G*_*A*_(*k*), *G*_*P*_(*k*)). We then chose, from the ensemble, the single exemplar network whose (${\tilde{g}}_{A}\left(k\right),\;{\tilde{g}}_{P}\left(k\right)$ ) was closest to the average (*G*_*A*_(*k*), *G*_*P*_(*k*)), [we minimized $\sum_{k}\left[\left|{\tilde{g}}_{A}\left(k\right)-G_{A}\left(k\right)\right|+\left|{\tilde{g}}_{P}\left(k\right)-G_{P}\left(k\right)\right|\right]$] and used that single network to represent a manipulated version of AC in which the 299 interactions are between random pairs of species. The key feature here is that the single chosen network has degree distributions that are unremarkable, but different from the observed.

For our other two degree manipulations, we conserved the observed degree distribution of one guild, but randomized the other (by redistributing the elements in rows, or in columns of the binary ***M*** ^*AC*^). We again chose a single exemplar network whose degree distribution, for the randomized guild, was closest to the expected distribution for the ensemble of 10,000 random networks.

For each of our three exemplar networks, we ran 25,000 simulations using bSO, bDA, and bRW. We used only one randomized network from each ensemble deliberately, the better to focus on the effect of manipulating *g*_*A*_(*k*) or *g*_*P*_(*k*) or both on the robustness distribution *f*(*R*).

#### 2.7.2 Plant extinction rank and degree

To explore whether (for example) high‐degree plants tend to go extinct toward the beginning of a primary extinction sequence, we recorded the position in a sequence when each plant became extinct as its extinction rank (*r*), 1 ≤ *r *≤ *P*. We ran each extinction model 25,000 times, using binary and weighted versions of AC, and computed *h*(*r*), the distribution of extinction rank for each species generated by the simulations. We tested for correlation, using the Spearman coefficient, between a plant's median extinction rank (*r*_m_) and degree (*k*). By construction, *r*_m_ should be the same for all plant species under the SO model, but not necessarily under the DA or RW models, since avalanches and random walks may tend to select (or avoid) high‐degree nodes preferentially.

## 3 RESULTS

### 3.1 Varying the value of the threshold for secondary extinctions

**Figure 3:**
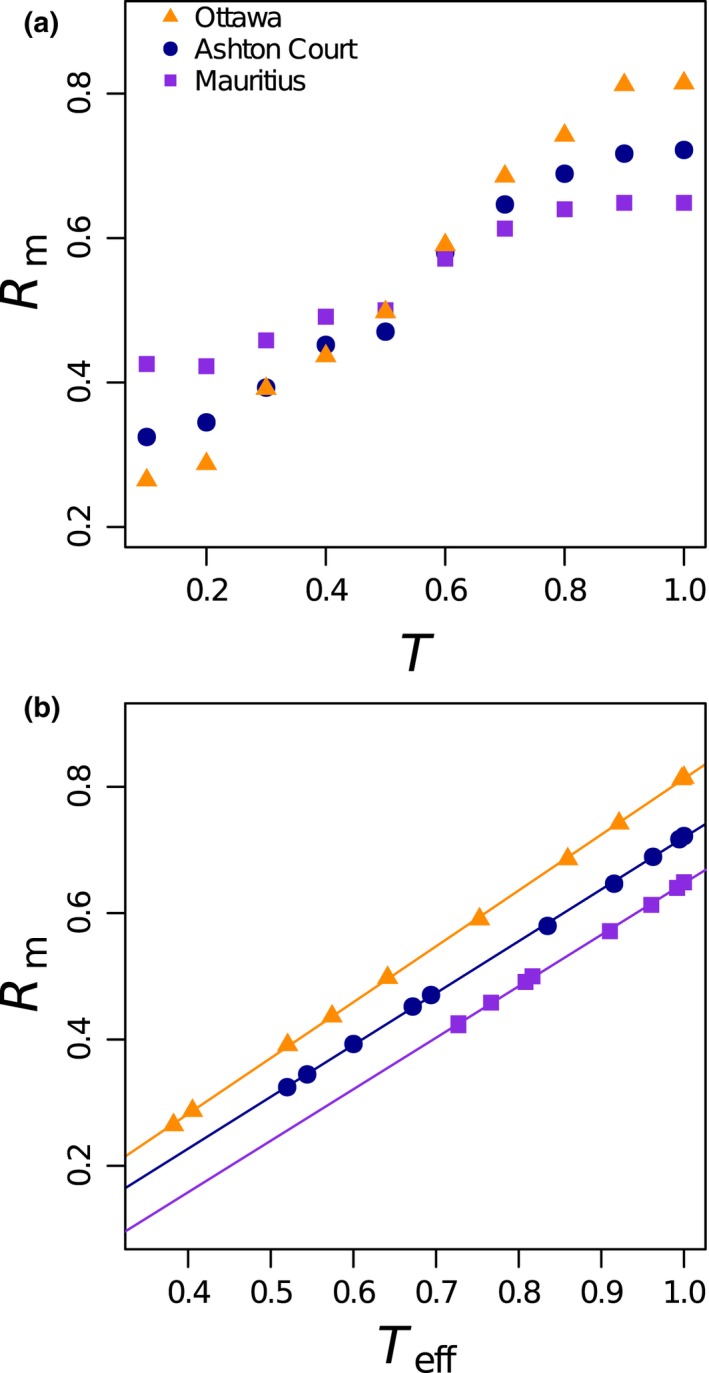
The relationship between extinction threshold (*T*), median robustness (*R*_m_), and effective threshold (*T*_eff_) for plant–pollinator networks: Ashton Court (triangles), Mauritius (squares), and Ottawa (circles) using the bSO model. Variation of (a) *R*_m_ with *T*, and (b) *R*_m_ with *T*_eff_

Median robustness *R*_m_ increases monotonically but nonlinearly with *T*. Figure 3a shows (for three illustrative networks) that there is a crossover; the least robust network at low *T* becomes the most robust at high *T*. This is an artifact of the variation of effective threshold with node degree; *R*_m_ increases linearly with *T*_eff_ and the three networks are increasingly robust in order of increased connectance, as found by Dunne et al. (2002), at all values of *T*_eff_ (Figure 3b for three illustrative networks; all six in Supporting information Figure S1). The remainder of our results are presented for the AC network where *T*_eff_ = 0.694 for our chosen *T *=* *0.5.

### 3.2 Robustness distributions for the Ashton Court network

**Figure 4:**
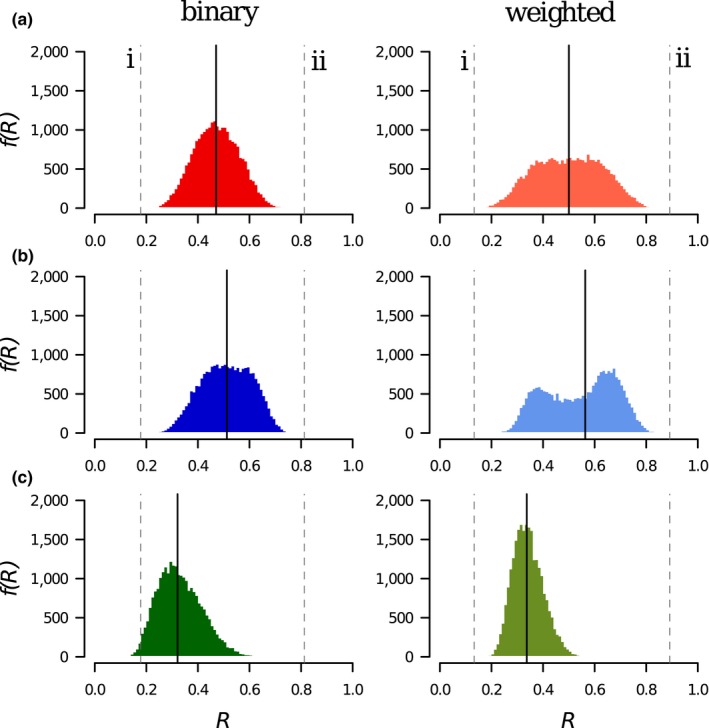
The distribution of robustness *f*(*R*) for the Ashton Court network, in binary (left column) and weighted (right column) form, generated by the three extinction models: (a) Secondary Only (SO), (b) Deterministic Avalanche (DA), and (c) Random Walk (RW). Median robustness *R*_m_ for each distribution indicated by the solid vertical line. (i) and (ii) indicate *R* values for the bSO model generated by removing plant species in increasing and decreasing degree order: b: 0.178 and 0.812, w: 0.133 and 0.891

The distributions *f*(*R*) produced by each of the three models for binary and weighted data using the Ashton Court network (Figure 4) are all rather broad, suggesting a strong dependence of *R* on the order in which plants are made extinct; the computed values span the range generated by primary extinction sequences in bSO with plants removed in increasing and decreasing order of degree (*R *=* *0.178 and *R *=* *0.812 respectively). The bSO model produces a relatively symmetrical *f*(*R*) with a median *R*_m_ **= 0.470. Using the bSO model as a baseline, the bDA model shifts *f*(*R*) to the right (Figure 4b: *R*_m_ = 0.512), inferring greater robustness, and bRW strongly shifts *f*(*R*) to the left (Figure 4c: *R*_m_ **= 0.337) inferring lower robustness. The same trends are shown for weighted data: *R*_m_ = 0.500 (wSO), 0.564 (wDA), and 0.321 (wRW).

### 3.3 Robustness distributions for other networks

The distributions *f*(*R*) for the other five networks tested (Supporting information Figures S2–S6) follow the same trends described above for Ashton Court. In every case, *R*_m_(DA) >* R*_m_(SO) >* R*_m_(RW) for both binary and weighted data. In general, distributions of robustness are broader for weighted data than binary data.

### 3.4 Effect of manipulating degree distribution

**Figure 5:**
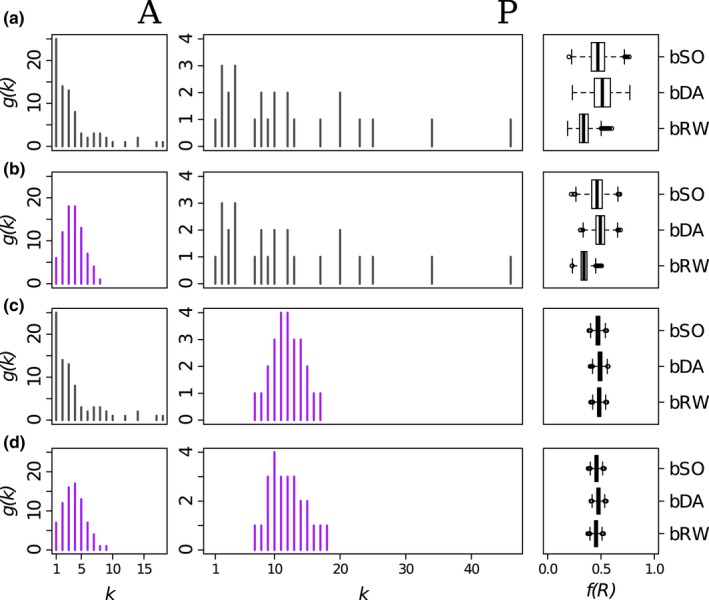
The effect of node degree distribution on robustness distribution *f*(*R*) for (a) the binary Ashton Court network and (b–d) manipulated networks as described in Section 2.7.1. Left column (A): pollinator degree distribution (gray—observed; purple—manipulated); central column (P): plant degree distributions; right column: summaries of *f*(*R*) from the bSO, bDA and bRW extinction models. [Box‐plots, with central lines showing median, boxes showing inter‐quartile range, and whiskers showing the 95% (2.5–97.5%) interval]

Compared to the results of the binary extinction models for the true AC network (Figure 5a), we found that narrowing the degree distributions caused the robustness distribution *f*(*R*) to be narrower (Figure 5b–d), and this was especially so when the plant degree distribution was manipulated (Figure 5c and d). This confirms that the observed, highly skewed, plant degree distribution of the AC network produces the broad robustness distributions we generate for this network. Note though that median robustness *R*_m_ remains in the same order (RW<SO<DA) in every case, showing the consistency of effect from these models.

### 3.5 Extinction rank of plant species, and the effect on *R*

**Figure 6:**
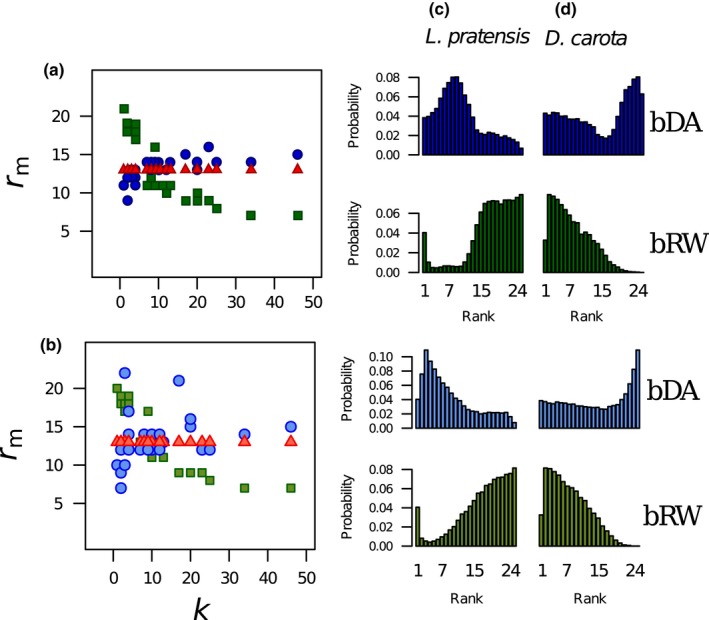
Variation of median extinction rank *r*_m_ with degree (*k*) for all 25 plant species in the Ashton Court network for the three extinction models (SO: red, DA: blue, and RW: green) and for (a) binary and (b) weighted edges. Spearman's rank correlation shows that all these associations are significant: positive for DA (blue) and negative for RW (green). Extinction rank distribution *h*(*r*) for two plant species (c) *Lathyrus pratensis* (*k *=* *2) and (d) *Daucus carota* (*k *=* *46) produced by the DA (blue) and RW (green) models

Plant degree is a predictor of the plant's extinction rank in the DA and RW models (Figure 6a and b). In the SO models, the rank should be constant for all plant species, irrespective of degree, because the extinction sequence is entirely random. In contrast, the observed extinction ranks of two example plant species from the DA and RW models are clearly skewed (Figure 6c and d). In the DA models, median extinction rank is positively correlated with plant degree (bDA: ρ = +0.803, *p* < 0.0001; wDA: ρ = +0.420, *p* = 0.03). For the RW models, *r*_m_ is negatively correlated with *k* (bRW: ρ = −0.960, *p* < 0.0001; wRW: ρ = −0.820, *p* < 0.0001). In other words, for the DA models, well‐connected plants are resistant to extinction; the model preferentially prunes the low degree plants so network robustness is high compared to the SO models (Figure 4b cf. Figure 4a). In contrast, in the RW models plants with high degree are more vulnerable to extinction (the model preferentially “homes in” on well‐connected plants) so network robustness is low compared to the SO models (Figure 4c cf. Figure 4a).

## 4 DISCUSSION

Robustness *R* is a valuable quantitative metric for describing and comparing the vulnerability of ecological networks to simulated extinctions. We confirm, through our framework of extinction models, that *R* is a consequence of both the model itself and the network structure. Our analysis reveals the mechanisms and fundamental network properties that drive observed trends in robustness.

Knockout extinction models that calculate robustness have been around for over a decade and the list of ecological rules they employ is growing. Building on the models of Memmott et al. (2004), Kaiser‐Bunbury et al. (2010) and Vieira and Almeida‐Neto (2015), we have brought together a suite of directly comparable knockout extinction models and applied them here to plant–pollinator networks. We have used an extinction threshold (pollinators can go extinct before all their plants go extinct and vice versa) that can be applied to all nodes. This addition has an ecological motivation—plants may decline to extinction due to reducing pollination (as modeled by Traveset et al., 2017), and adds greatly to the flexibility of the model. Having *T *<* *1 allows us to create weighted versions of our models and provides the potential for feedback between the trophic levels and, hence, avalanches of extinctions cascading across the network (e.g., as shown by Campbell et al., 2012 and Vieira & Almeida‐Neto, 2015). Cascades are more likely as *T* is decreased. We chose a middle value of *T* (0.5). The exact value chosen is not a vital ingredient of this work, but can make a big difference to mean robustness (Figure 3, Supporting information Figure S1). We therefore recommend that researchers test at least the qualitative robustness of their conclusions to varying values of threshold.

All our extinction models, in binary and weighted form, produce a broad distribution of robustness values *f*(*R*) for each network that we analyzed, indicating that there are aspects of the structure of the network that cause this variation. We found the degree distribution of the plants, in particular, to be an important driver of robustness variation. Plant–pollinator networks tend to have fewer plant species than pollinator species (*P *< *A*), so the potential for a skewed plant degree distribution is greater, thus making it more influential on robustness in our test network (Memmott, 1999). Of the six networks we analyzed, those that have one particularly highly connected plant (Ashton Court—Figure 4, and Hickling—Supporting information Figure S5) have the broadest *f*(*R*); those with a more homogenous plant degree distribution are narrower. We note in passing that the largest plant degree is strongly correlated with nestedness in these networks (Supporting information Figure S7).

Though “robustness” has in the past been used to suggest priorities for conservation or management (Devoto, Bailey, Craze, & Memmott, 2012; Pocock et al., 2012), extinction models are not an attempt to predict precisely how an ecosystem would collapse. They do, nonetheless, offer a means to quantify and compare the structure of ecological networks, but to do this we need to ensure we are comparing like‐for‐like.

Plant–pollinator communities are increasingly described with weighted interactions. We found (Figure 4, Supporting information Table S1) that introducing weighted interactions has the effect of amplifying the outcomes observed for binary data: the inter‐quartile range of the robustness distribution *f*(*R*) increases in all models for weighted networks, and the shifts in median robustness for DA and RW compared to SO are larger. Weights tend to increase the skew of the plant degree distribution because high‐degree species accumulate high edge weights and low degree species only gain a small fraction of the overall weight in the network. This exaggerates effects in *f*(*R*) and highlights the importance of including interaction weights in robustness analysis, and in exploring all of the distribution *f*(*R*), not just its central tendency. Future work should continue to explore the full effects of weighted data.

There are different ways in which extinction models can use feedback between trophic levels and we developed two illustrative models: the Deterministic Avalanche (DA) and the Random Walk (RW) models. These models (and others like the cascade model developed by Vieira & Almeida‐Neto, 2015) may appear to be generating new outcomes, but in reality, they simply produce a nonrandom sample of robustness values from those generated by a simple SO model. The AC dataset generated a very wide range of *R* values, all of which can be realized in the SO models. The DA and RW models preferentially sample extinction sequences to produce skewed subsets of the SO outcomes (the *P*! extinction sequences are not all equally likely, and some will be impossible). The DA Model preferentially samples nodes that are 1 step away from each other in the network and extinctions can “ripple out” from each trigger. In some cases, the DA model produces a double‐peaked *f*(*R*) distribution. This corresponds to networks where the highest plant degree, as a fraction of the number of pollinators, is large—the Ashton Court and Hickling networks for example. In contrast to DA, in the RW model plant extinctions tend to jump from plant to plant away from a trigger. Although both the DA and RW models are ecologically credible, they produce opposing results, demonstrating the influence of the model on the assessment of robustness. It is important for researchers using robustness models to have a clear justification for the model they use, and a clear understanding of how much their results are influenced by the model as well as the network data.

All of these extinction models are designed to be applied to real ecological network data. Therefore, it is vital to consider the quality and reliability of the data being used. Empirical pollination networks vary hugely in sampling method, period of collection and taxonomic resolution, all of which can affect metrics of network structure. Factors such as relative species abundance and time of sampling can lead to over‐ or underestimating the degree of a plant species in a network (e.g., Blüthgen et al., 2006).This will affect the outcomes of knock‐on extinction models and could easily over‐ or underestimate the robustness and the importance of particular plant species. We caution against comparing the outcomes of extinction models across multiple networks, for example, in meta‐analyses or comparative analyses, without consideration of the data and the methods used to collect them. CaraDonna et al. (2017) highlight the potential pitfalls of assuming that a network constructed by aggregating samples over time is an appropriate representation of a community. Further work in understanding temporal variation and the description of fully resolved plant–pollinator networks is key to improving the utility of extinction models.

Current robustness models still lack the biological realism needed to make reliable ecological predictions. They are, however, useful for understanding and separating the effects of mechanism and network structure. We recommend therefore that researchers seeking greater ecological realism in models pay due attention to the details of the models themselves. Ecological conclusions drawn from robustness models may become less surprising when model developments are taken into account. We hope that by improving our understanding of extinction models at a mechanistic level and by setting out different areas of model extension, our work will guide future developments in the analysis of the vulnerability of ecosystems to environmental change.

## CONFLICT OF INTEREST

None declared.

## DATA ACCESSIBILITY

This work has used the Web of Life database to access ecological network data: http://www.web-of-life.es. All scripts used to run our suite of models are available as supporting material for this paper.

## AUTHORS’ CONTRIBUTIONS

All authors designed the methodology, discussed the results, and commented on the manuscript at all stages. MSB coded the models and analyzed the data with technical advice and support from RJ and MJOP. MSB drafted the manuscript. All authors contributed critically to the drafts and gave final approval for publication.
