# Supplementary material for: Effects of model choice, network structure, and interaction strengths on knockout extinction models of ecological robustness
Source: Ecol Evol. 2018 Oct 31;8(22):10794–804. doi: 10.1002/ece3.4529 (PMC6262911; doi:10.1002/ece3.4529)
Supplement: Supplementary file 1 [file ECE3-8-10794-s001.pdf]

Supporting Information

*Effects of model choice, network structure and interaction strengths on knockout extinction models of ecological robustness*

Bane et al. 2018

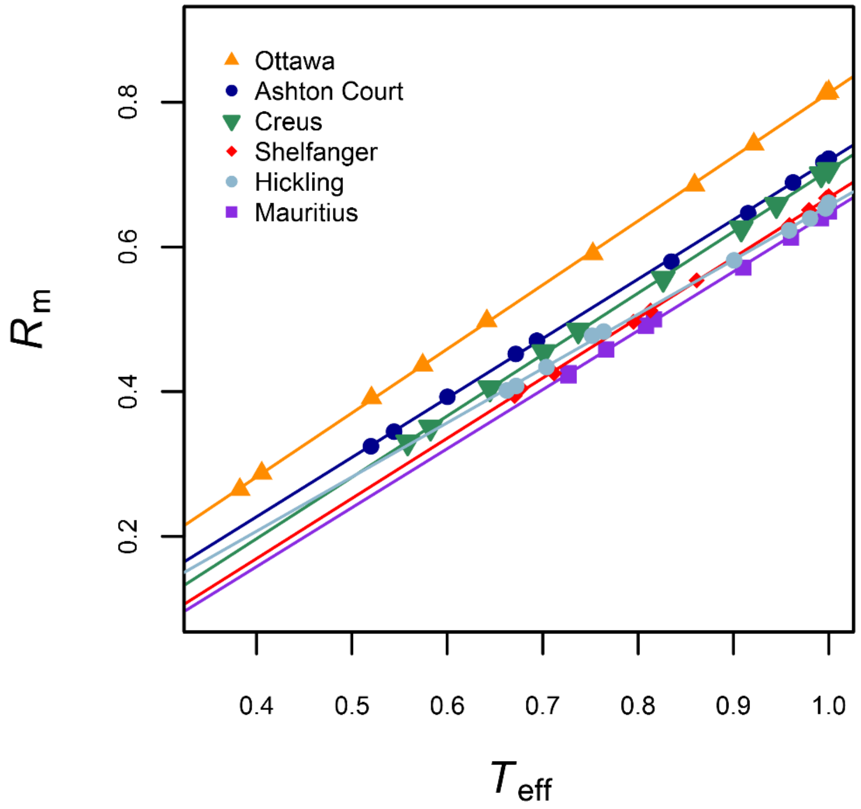


**Figure S1:** The relationship between $T_{eff}$ for the 6 networks (summarised in Table 1) used in this paper. All networks show a linear trend – lines are plotted to guide the eye. All are near parallel with the exception of Hickling which lies very close to Shelfanger and crosses in the middle – the $R_m$ values for Hickling are less representative as the $f(R)$ distributions are so broad.

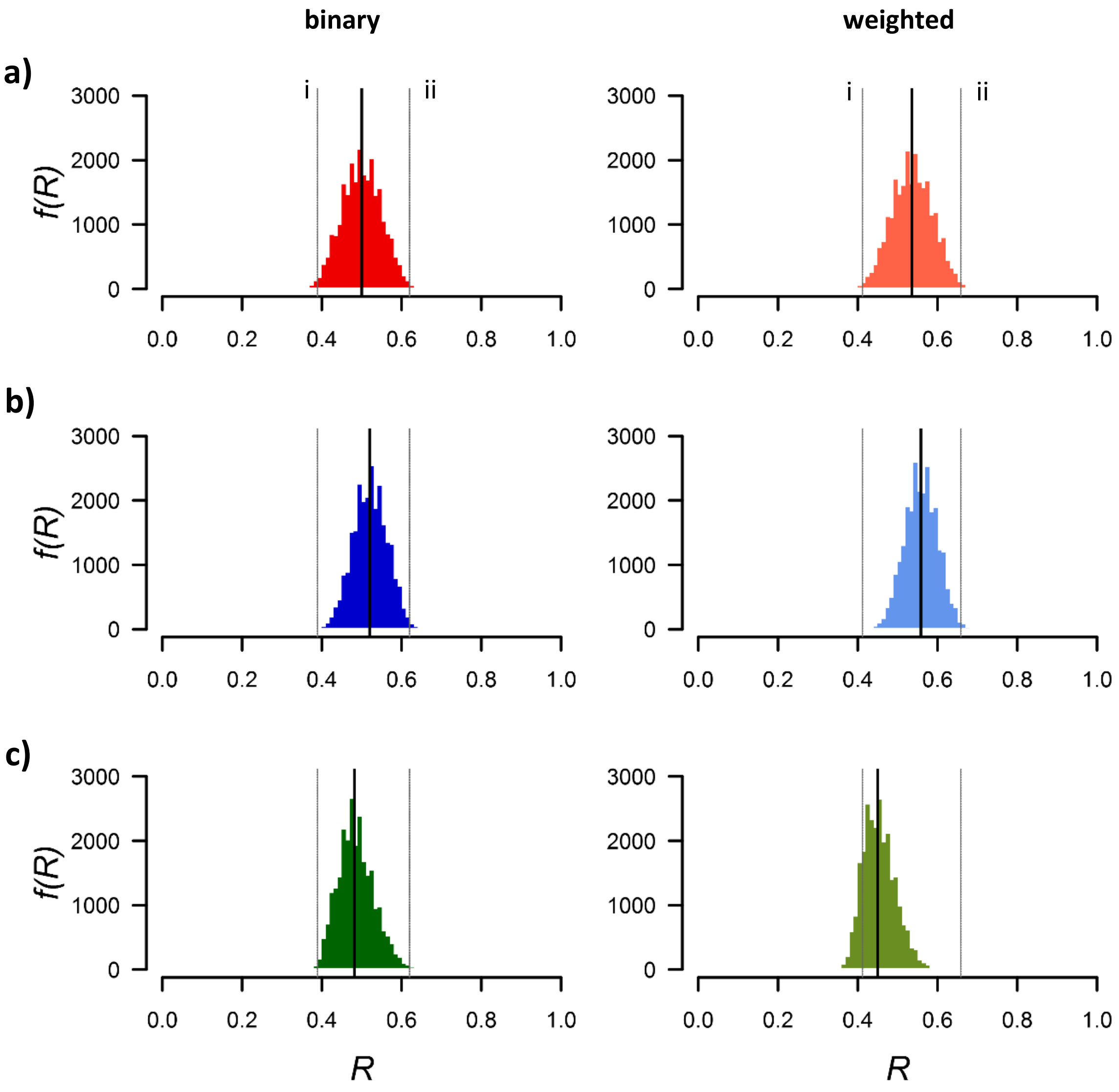


**Figure S2:** The distribution of robustness $f(R)$ for the Ottawa network, binary (left column) and weighted (right column), generated by the 3 extinction models: **(a)** Secondary Only (SO), **(b)** Deterministic Avalanche (DA) and **(c)** Random Walk (RW). Median robustness $R_m$ for each distribution is indicated by the black vertical line. Thin, grey lines indicate $R$ values for the SO model when plants are removed in increasing; (i) b:0.389, w:0.412; and decreasing order (ii); b:0.620, w:0.658. The Ottawa $f(R)$ distributions are not particularly broad. $R_m$(DA) > $R_m$(SO) > $R_m$(RW). The observed breadth and shifts correspond to the homogeneity of the network; the plant degree distribution is less skewed than other plant-pollinator networks. All Ottawa $f(R)$ distributions cross (i) and (ii), particularly so for wRW. This is not surprising as many plant nodes have the same degree and therefore there are many node sequences in degree order – (i) and (ii) are not unique.

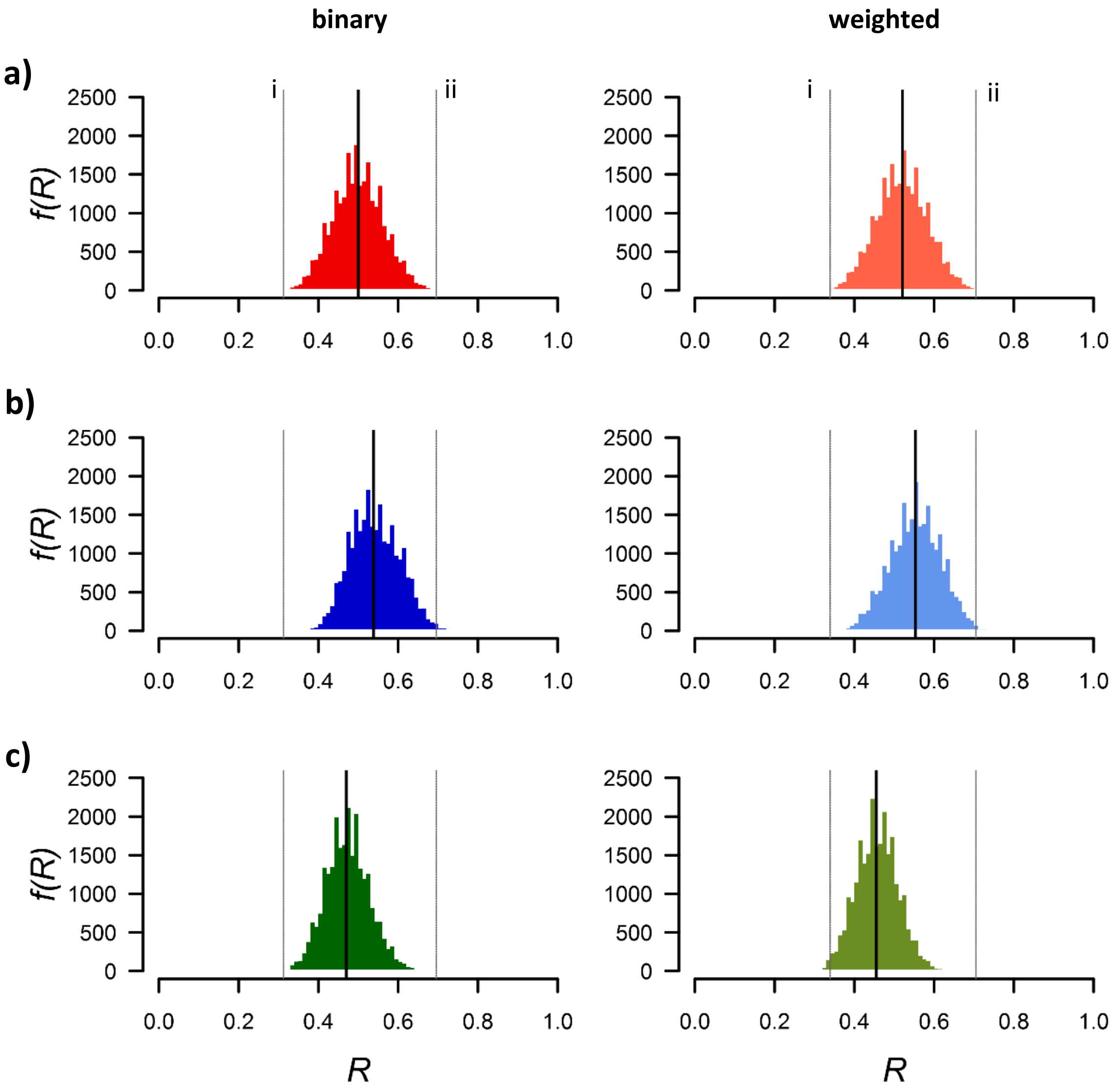


**Figure S3:** The distribution of robustness $f(R)$ for the Mauritius network, binary (left column) and weighted (right column), generated by the 3 extinction models: **(a)** Secondary Only (SO), **(b)** Deterministic Avalanche (DA) and **(c)** Random Walk (RW). Median robustness $R_m$ for each distribution is indicated by the black vertical line. Thin, grey lines indicate $R$ values for the SO model when plants are removed in increasing (i); b:0.314, w:0.339; and decreasing order (ii); b:0.696, w:0.705. The Mauritius $f(R)$ distributions are quite broad. $R_{\mathrm{m}}(\mathrm{DA}) > R_{\mathrm{m}}(\mathrm{SO}) > R_{\mathrm{m}}(\mathrm{RW})$. The observed breadth and size of shifts correspond to the homogeneity of the network.

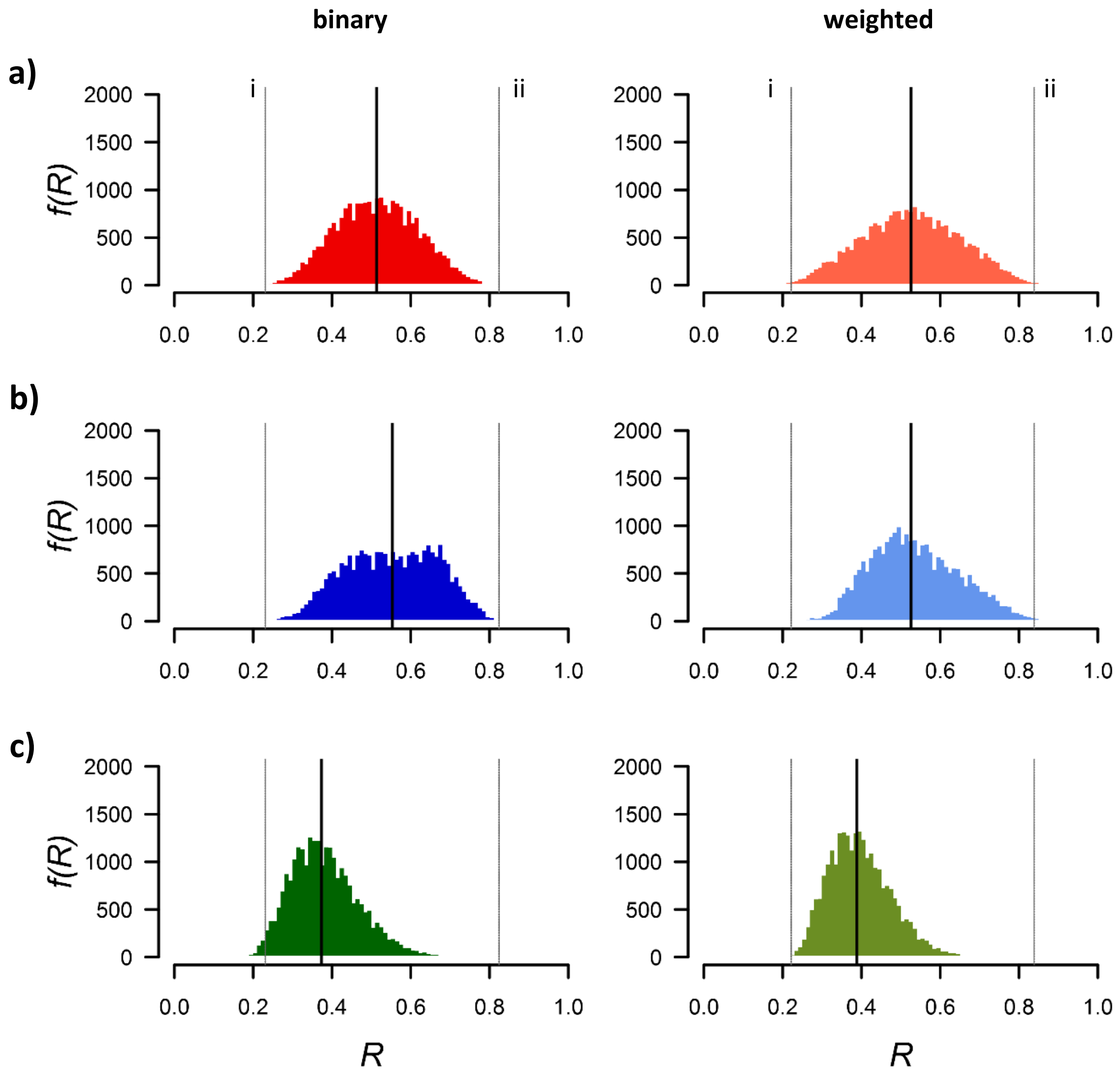


**Figure S4:** The distribution of robustness $f(R)$ for the Shelfanger network, binary (left column) and weighted (right column), generated by the 3 extinction models: **(a)** Secondary Only (SO), **(b)** Deterministic Avalanche (DA) and **(c)** Random Walk (RW). Median robustness $R_m$ for each distribution is indicated by the black vertical line. Thin, grey lines indicate $R$ values for the SO model when plants are removed in increasing (i); b:0.231, w:0.222; and decreasing order (ii); b:0.825, w:0.839. The Shelfanger $f(R)$ distributions are broad. $R_{\mathrm{m}}(\mathrm{DA}) \gtrsim R_{\mathrm{m}}(\mathrm{SO}) > R_{\mathrm{m}}(\mathrm{RW})$. Note the left skewed peak for wDA (though $R_{\mathrm{m}}(\mathrm{DA}) \gtrsim R_{\mathrm{m}}(\mathrm{SO})$). The breadth and size of shifts correspond to the homogeneity of the network.

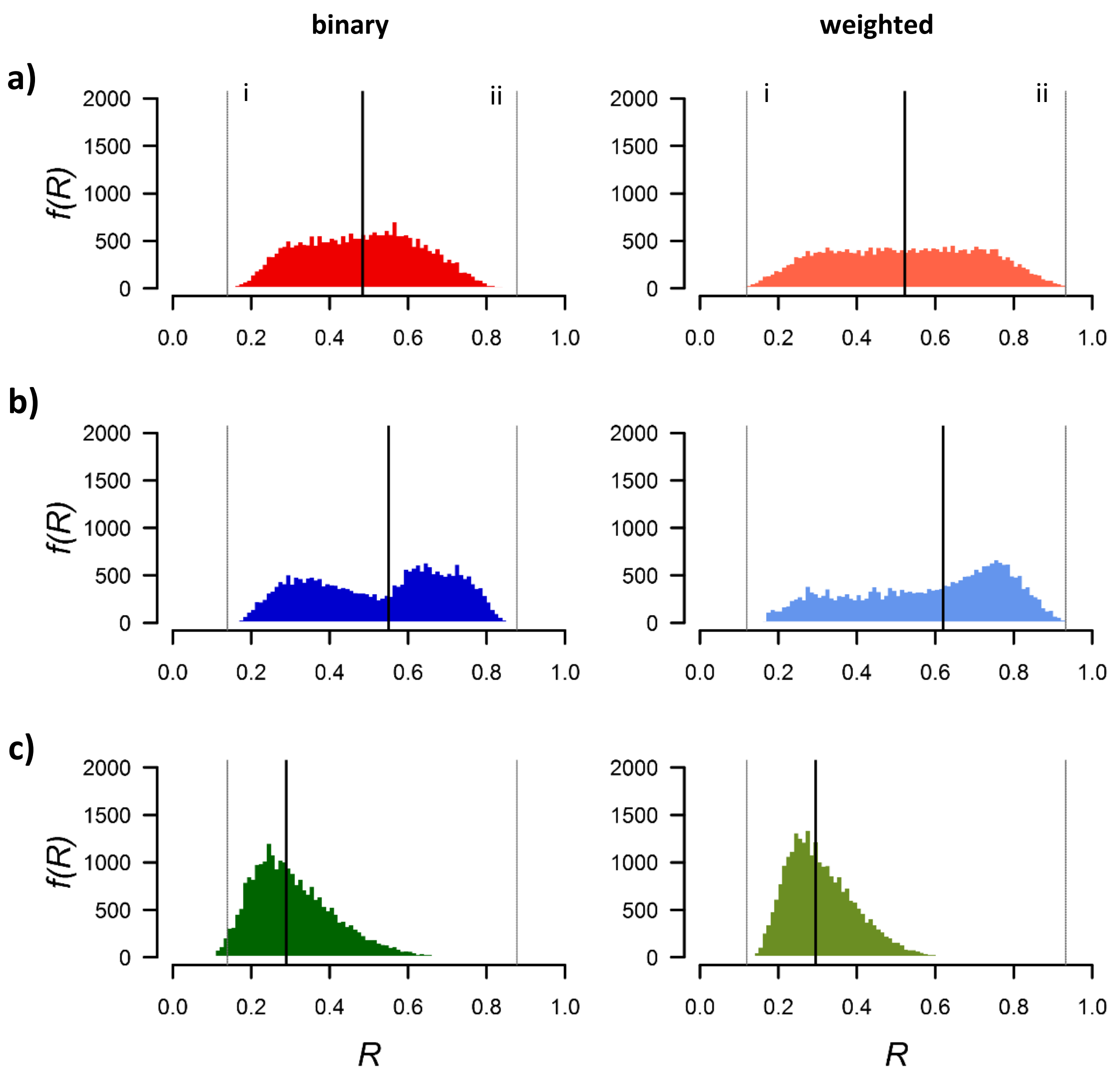


**Figure S5:** The distribution of robustness $f(R)$ for the Hickling network, binary (left column) and weighted (right column), generated by the 3 extinction models: **(a)** Secondary Only (SO), **(b)** Deterministic Avalanche (DA) and **(c)** Random Walk (RW). Median robustness $R_m$ for each distribution is indicated by the black vertical line. Thin, grey lines indicate $R$ values for the SO model when plants are removed in increasing (i); b:0.140, w:0.120; and decreasing order (ii); b:0.8775, w:0.9325. The Hickling $f(R)$ distributions are quite broad. $R_{\mathrm{m}}(\mathrm{DA}) > R_{\mathrm{m}}(\mathrm{SO}) > R_{\mathrm{m}}(\mathrm{RW})$. The very broad distributions, with strong shifts between models correspond to the highly skewed plant degree distribution and network heterogeneity.

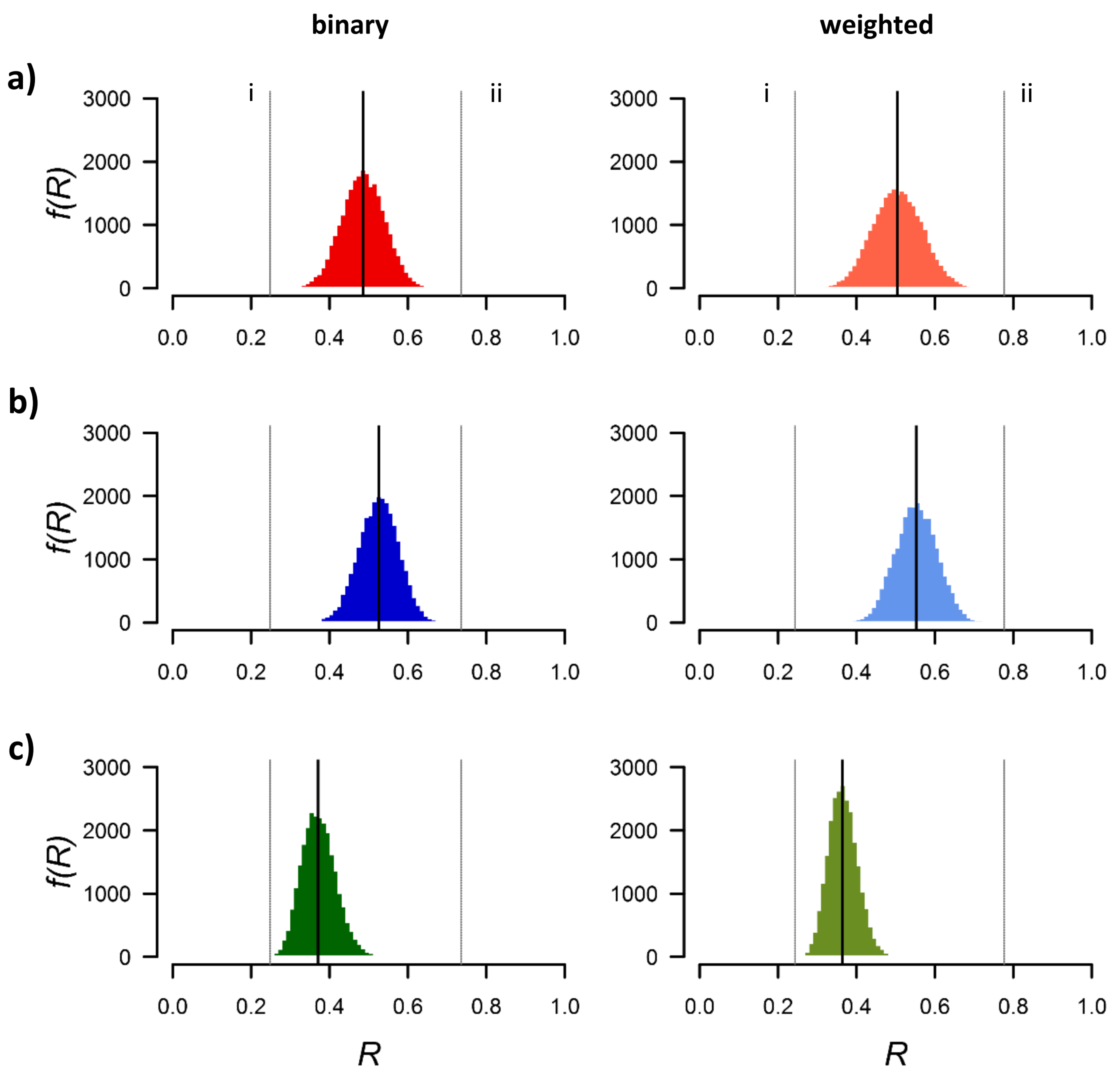


**Figure S6:** The distribution of robustness $f(R)$ for the Creus network, binary (left column) and weighted (right column), generated by the 3 extinction models: **(a)** Secondary Only (SO), **(b)** Deterministic Avalanche (DA) and **(c)** Random Walk (RW). Median robustness $R_m$ for each distribution is indicated by the black vertical line. Thin, grey lines indicate $R$ values for the SO model when plants are removed in increasing (i); b:0.249, w:0.243; and decreasing order (ii); b:0.736, w:0.777. The Creus $f(R)$ distributions are quite broad. . $R_m(DA) > R_m(SO) > R_m(RW)$. The observed breadth and shifts correspond to the homogeneity of the network. All Creus $f(R)$ distributions sit well within (i) and (ii), indicating that degree order sequences are unique cases of extreme robustness values and unlikely to occur in 25,000 simulations.

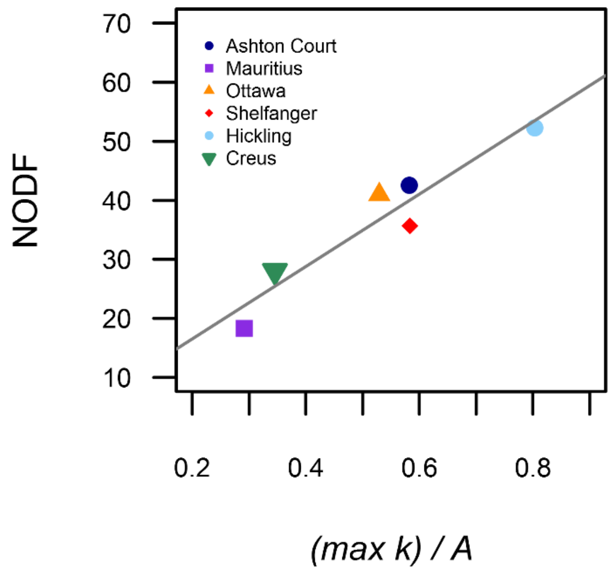


**Figure S7:** Correlation between the normalised, largest plant degree *(max k)/A*, and nestedness (NODF) (Almedia-Neto *et al.* 2008). Spearman's rank correlation: $\rho = +0.83$, $p = 0.04$. Line plotted to guide the eye.

| **Network** | **Plants** (*P*) | **Pollinators** (*A*) | **Interactions** (*E*) | **Connectance** (*c*) | **Nestedness** (NODF) | **Median R ($R_m$) Secondary Only (bSO)** | **Median R ($R_m$) Deterministic Avalanche (bDA)** | **Median R ($R_m$) Random Walk (bRW)** | **$R_m$ for plant extinctions in increasing degree order** | **$R_m$ for plant extinctions in decreasing degree order** | **$T_{eff}$ at $T = 0.5$** |
|---|---|---|---|---|---|---|---|---|---|---|---|
| Ashton Court | 25 | 79 | 299 | 0.151 | 42.54 | 0.470 | 0.512 | 0.337 | 0.178 | 0.812 | 0.694 |
| Ottawa | 13 | 34 | 141 | 0.319 | 40.96 | 0.500 | 0.520 | 0.450 | 0.389 | 0.620 | 0.642 |
| Mauritius | 14 | 24 | 46 | 0.137 | 18.30 | 0.500 | 0.539 | 0.455 | 0.312 | 0.696 | 0.817 |
| Shelfanger | 16 | 36 | 85 | 0.148 | 35.66 | 0.514 | 0.554 | 0.389 | 0.231 | 0.825 | 0.813 |
| Hickling | 17 | 61 | 146 | 0.141 | 52.27 | 0.485 | 0.551 | 0.295 | 0.140 | 0.878 | 0.764 |
| Creus | 32 | 81 | 319 | 0.123 | 28.01 | 0.486 | 0.526 | 0.364 | 0.249 | 0.736 | 0.737 |

**Table S1 (a):** Summary of results for all networks in this paper in binary form

| **Network** | **Plants** (*P*) | **Pollinators** (*A*) | **Interactions** (*E*) | **Connectance** (*c*) | **Nestedness** (NODF) | **Median R ($R_m$) Secondary Only (wSO)** | **Median R ($R_m$) Deterministic Avalanche (wDA)** | **Median R ($R_m$) Random Walk (wRW)** | **$R_m$ for plant extinctions in increasing degree order** | **$R_m$ for plant extinctions in decreasing degree order** |
|---|---|---|---|---|---|---|---|---|---|---|
| Ashton Court | 25 | 79 | 2183 | 0.151 | 42.54 | 0.500 | 0.564 | 0.321 | 0.133 | 0.891 |
| Ottawa | 13 | 34 | 992 | 0.319 | 40.96 | 0.536 | 0.559 | 0.482 | 0.412 | 0.658 |
| Mauritius | 14 | 24 | 112 | 0.137 | 18.30 | 0.521 | 0.553 | 0.470 | 0.339 | 0.705 |
| Shelfanger | 16 | 36 | 980 | 0.148 | 35.66 | 0.526 | 0.526 | 0.373 | 0.222 | 0.839 |
| Hickling | 17 | 61 | 3150 | 0.141 | 52.27 | 0.523 | 0.620 | 0.289 | 0.120 | 0.933 |
| Creus | 32 | 81 | 1227 | 0.123 | 28.01 | 0.505 | 0.553 | 0.371 | 0.243 | 0.777 |

**Table S1 (b):** Summary of results for all networks in this paper in weighted form
